# Description of eight new mitochondrial genomes for the genus *Neoarius* and phylogenetic considerations for the family Ariidae (*Siluriformes*)

**DOI:** 10.5808/gi.23059

**Published:** 2023-12-29

**Authors:** Luiz Guilherme Pereira Pimentel, Iuri Batista da Silva, Igor Henrique Rodrigues-Oliveira, Rubens Pasa, Fabiano Bezerra Menegídio, Karine Frehner Kavalco

**Affiliations:** 1 Laboratory of Ecological and Evolutionary Genetics, Federal University of Viçosa, Rio Paranaíba, Minas Gerais 38810-000, Brazil; 2 Laboratory of Bioinformatics and Genomics, Federal University of Viçosa, Rio Paranaíba 38810-000, Brazil; 3 Graduate Program in Zoology, Federal University of Minas Gerais, Belo Horizonte 31270-901, Brazil; 4 Integrated Biotechnology Center, University of Mogi das Cruzes, Mogi das Cruzes, SP 08780-911, Brazil; 5 Technological Research Center, University of Mogi das Cruzes, Mogi das Cruzes, SP 08780-911, Brazil

**Keywords:** marine catfish, mitogenomes, phylogeny

## Abstract

The genus *Neoarius*, known as marine catfish, is a group of the family Ariidae, composed of 10 species found in Oceania. None of the species in this genus have their mitochondrial genome described, which is highly valuable in phylogenetic and molecular evolution studies. For the present work, eight species from the *Neoarius* genus were selected: *Neoarius utarus*, *Neoarius midgleyi*, *Neoarius graeffei*, *Neoarius leptaspis*, *Neoarius berenyi*, *Neoarius paucus*, *Neoarius pectoralis*, and *Neoarius* aff. *graeffei*. DNA sequences of the eight species were obtained through the NCBI Sequence Read Archive (SRA) database, and the mitochondrial genomes were assembled using the NOVOplasty tool on the Galaxy platform, subsequently annotated with the MitoAnnotator tool. We then utilized the protein-coding genes from the mitogenomes to estimate the phylogenetic relationships within the group, including seven additional mitogenomes available in the NCBI. In all species, the mitochondrial genomes presented 13 protein-coding genes, 2 rRNA genes, 22 tRNA genes, and 1 D-loop.

## Introduction

*Neoarius* is a genus of catfish belonging to the family Ariidae within the order Siluriformes. Currently, it comprises 11 described species distributed in Australia and New Guinea, with conservation status ranging from "least concern" to "vulnerable" according to the International Union for Conservation of Nature Red List (IUCN) [1].

Among the 11 species that make up the Neoarius genus, six species occur in marine environments, namely *Neoarius graeffei*, *Neoarius leptaspis*, *Neoarius pectoralis*, *Neoarius berneyi*, *Neoarius paucus* while the others, such as *Neoarius utarus*, *Neoarius midgleyi*, *Neoarius utarus*, *Neoarius latirostris*, *Neoarius coatesi*, *Neoarius taylori*, and *Neoarius velutinus*, inhabit freshwater environments [2]. The reproductive period for these species typically starts in spring, around September, and extends until the end of summer, in February. Notably, *N. graeffei* employs a rare reproductive strategy known as mouthbrooding, where the eggs are incubated in the mouth of the adult individual until they mature enough to be independent [3].

The catfish of the Ariidae family have a wide distribution across the globe, being found in various regions and countries such as Brazil, Australia, and New Guinea. They inhabit coastal, estuarine, and large river regions in both tropical and temperate areas. The majority of species have a coastal distribution, but there are also exclusively marine species found at various depths, as well as species occurring in freshwater environments. In species from marine habitats, males exhibit a unique behavior of mouthbrooding, where they incubate the eggs in their mouths. This characteristic sets them apart within the Ariidae family. Currently, the family comprises 156 species distributed among 30 genera.

The mitochondrial genome, also known as the mitogenome, consists of a circular extrachromosomal DNA molecule present in the mitochondria. In eukaryotic organisms, the mitochondrial genome has an average size of 16–17 kbp and contains highly conserved genes. In vertebrates, there are a total of 37 genes, including 13 protein-coding genes (PCGs), two rRNA genes, and 22 tRNA genes necessary for the translation of proteins encoded by mitochondrial DNA, in addition to the control region known as the D-loop. Comparing mitochondrial genomes from different groups allows for evolutionary and phylogenetic studies, as there is significant similarity among mitogenomes in closely related taxa. This similarity enables researchers to trace the evolutionary history and relationships between species and understand their genetic diversification over time [4].

The mitochondrial genome in fishes serves various purposes, such as phylogenetic reconstruction, phylogeography, population migration observation, geographic distribution analysis, genetic diversity analysis among distinct populations, examination of gene order variations, haplotype variations, and gene flow patterns. Therefore, conducting studies involving the mitochondrial genome in fishes, as well as other vertebrate groups, is of great importance [4].

The objective of this study is to describe the mitochondrial genome of eight out of the 11 existing species within the *Neoarius* genus, as none of the species currently have their mitogenome characterized. To achieve this, we assembled the mitogenomes of *Neoarius berenyi*, *Neoarius utarus*, *Neoarius midgleyi*, *Neoarius graeffei*, *Neoarius utarus*, *Neoarius leptaspis*, *Neoarius paucus*, and *Neoarius* aff. *graeffei*. Additionally, we conducted a phylogenetic analysis using the PCGs from the assembled mitogenomes, providing new insights into the interspecific phylogenetic relationships within Neoarius.

## Methods

For the completion of this study, DNA samples from *Neoarius* species were collected from muscular tissue and sequenced by Iridian Genomics In platform HiSeq x Ten of Illumina. The resulting sequence data were provided by Dr. Ricardo Betancur through Sequence Read Archive (SRA) files hosted on NCBI (Table 1). These files were imported into the Galaxy Europe platform [5]. Subsequently, we conducted mitochondrial genome assembly using NOVOplasty v4.2 with a K-mer size of 39 [6]. We utilized sequences from the cytochrome B oxidase gene of the respective species available on GenBank (Table 1) as seeds for the assembly. The circularized sequences were then annotated using the MitoAnnotator software on the MitoFish server [7]. Finally, a comparative analysis was conducted using BLAST among all *Neoarius*, *Arius*, *Hypostomus*, and *Occidentarius* mitogenomes included in our study, along with our assembly of *N. graeffei*, using the BRIG software [8].

For phylogenetic reconstruction, we manually extracted all 13 PCGs from the eight *Neoarius* mitogenomes, as well as the PCGs from ten other Siluriformes species available on GenBank (Supplementary Material 1). Individual PCGs were aligned using MEGA 11 software [9] with the MUSCLE algorithm [10]. Subsequently, alignments were concatenated using SequenceMatrix v1.7.8 software [11]. Phylogeny was generated using IQ-TREE web server software [12], with parameters of 10,000 replicates of Ultrafast Bootstrap iterations and replicates. Tree visualization was performed using the Interactive Tree of Life (IToL) online tool [13].

## Results and Discussion

**Fig. 1.**
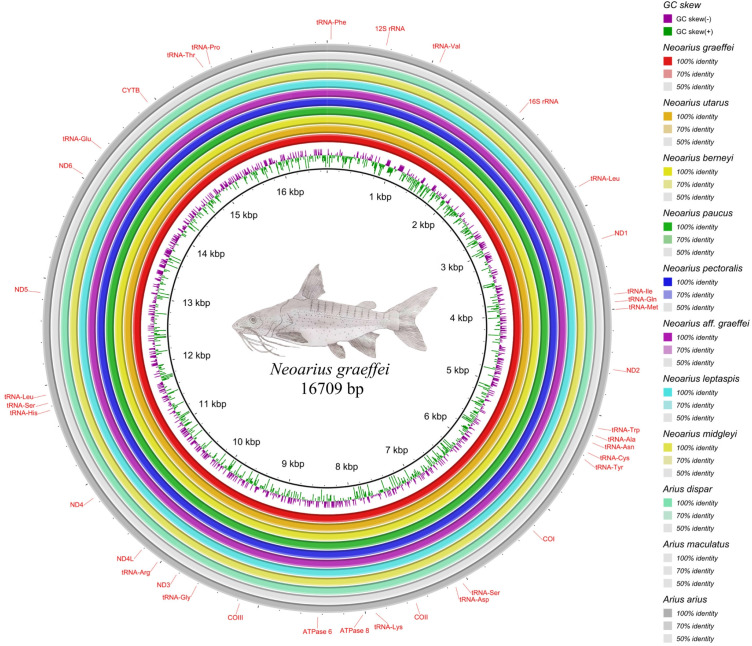
Comparison of the eight mitochondrial genomes of the genus *Neoarius* with the mitochondrial genomes of the species used as outgroups in the phylogenetic reconstruction. The mitogenomes of the ingroup are located on the inner part of the circle, while the outgroup is located on the outgroup.

We observed that the mitochondrial genomes have similar characteristics among them. All mitogenomes presented 22 tRNA genes, 13 PCGs, 2 rRNA genes, and a control region called the D-loop, which is consistent with other groups within the same family and even what is typically expected in vertebrates (Fig. 1) [4,14-16]. The size of the mitochondrial genomes was also similar, with 16,709 bp for *N. berneyi*, *N. graeffei*, *N. leptaspis*, *N. paucus*, and *N. midgleyi*, 16,702 bp for *N. utarus*, 16,710 bp for *N.* aff. *graeffei* RB-2021, and 16,711bp for *N. pectoralis* (Supplementary Material 2).

The complete mitochondrial genomes of *N. berenyi*, *N. midgleyi*, *N. leptaspis*, *N.* aff. *graeffei*, and *N. paucus* showed a CG composition of 45%, while *N. utarus*, *N. pectoralis*, and *N. graeffei* presented 44% of CG content. These calculations were performed using the geecee tool [17]. The largest gene in the mitochondrial genome was ND5, occupying 5.48% of the entire mitogenome for all species, with a variation of 913 bp and 914 bp.

**Fig. 2.**
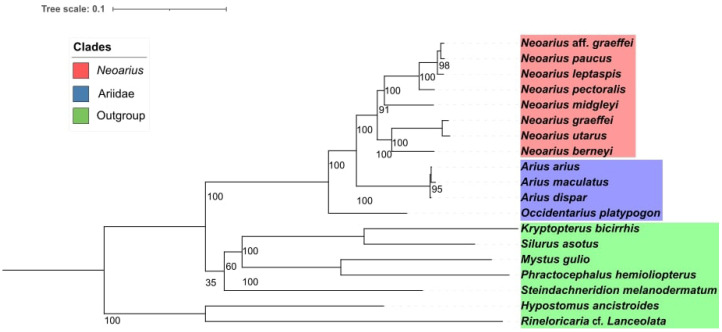
Phylogeny visualized in Interactive Tree of Life (IToL), with species forming the internal group (target group), with the eight species of *Neoarius* shown in red, followed by the genera *Arius*, which is the sister genus of *Neoarius*, and by the genus *Occidentarius*, being the most distant genus of the Ariidae family from Neoário. And shown in green are the siluriformes from other phylogenetically more distant families of Ariidae, being composed of the genera, *Hypostomus*, *Kryptopterus*, *Phractocephalus*, *Silurus*, *Steindachneridion*, and *Rineloricaria*.

The phylogeny demonstrated a monophyletic grouping among *Neoarius* species, which could be separated into two distinct clades. One of these clades showed *N. berneyi* as the sister group to the clade formed by *N. graeffei* and *N. utarus*, while in the other clade, we observed, *N. midgleyi* as the first species to diverge, followed by *N. pectoralis*, and with *N. leptaspis* as the sister group to the clade containing *N. paucus* and *N.* aff. *graeffei* (Fig. 2). All internal branches of *Neoarius* showed high bootstrap values (>90). An interesting observation in our study was that the mitogenomes from the libraries identified as *N.* aff. *graeffei* and *N. graeffei* did not group together and, in fact, each of them fell into one of the two *Neoarius* subclades, indicating that both samples were extracted from different species within the genus.

Few studies have a complete phylogeny of the genus; however, from the limited number of species present in the phylogenetic relationships, we can make some comparisons with the phylogeny observed in the present study, as in the work of Betancur [18]. In the work of Betancur [18], a phylogenetic reconstruction of the family Ariidae was conducted using molecular data, including the *cytochrome b*, *ATP synthase subunit 6* and *8*, *12S* and *16S ribosomal* genes, and the nuclear *rag2* gene. It was observed that *N. graeffei* aligned closely to *N. berenyi*, but between these two species, it aligned with *N.* aff. *graeffei*. However, when considering the overall phylogenetic relationship, the grouping of these two species remains quite similar, with *N. utarus* included in the same clade [18].

Another work involving phylogenetic reconstruction of the *Neoarius* genus is the study by Barathkumar and Thangaraj [19]. In the study by Barathkumar and Thangaraj [19], molecular data, specifically the cytochrome oxidase 1 gene, was used to conduct the phylogenetic reconstruction of the families Ariidae, Bagridae, and Plotosidae. Among the *Neoarius* genus, only *N. midgleyi* and *N. graeffei* were present in the study. The phylogenetic relationship revealed a grouping between these two species. Additionally, some species from the *Arius* genus were also included in the study, but there was no alignment of *Neoarius* and *Arius* in the same clade [19].

## Conclusion

The mitochondrial genomes of the studied species showed many similarities in terms of size, composition, and nucleotide percentage. Despite their stable organization, the mitogenomes proved to be valuable in understanding the molecular evolution of these eight species belonging to the *Neoarius* genus. New studies using other sources of data, such as phylogenies based on nuclear loci or even the assembly of mitochondrial genomes from the remaining species, can be applied to fill possible gaps in the knowledge of the group's evolution and gain a better understanding of the speciation process.

## Figures and Tables

**Table 1.** With all the access data for DNA sequences and seeds in NCBI that were used in the assembly of the mitochondrial genomes described in the present study

Species	SRA	BioProject	Accession No. Seed	SRA Ref.
*Neoarius utarus*	SRR14639415	PRJNA728311	FJ626209.1	Iridian genomes
*Neoarius graeffei*	SRR14629392	PRJNA728331	FJ013173.1	Iridian genomes
Neoarius midgleyi	SRR14627802	PRJNA728303	FJ626227.1	Iridian genomes
*Neoarius leptaspis*	SRR14646458	PRJNA728298	FJ626224.1	Iridian genomes
*Neoarius berneyi*	SRR14608389	PRJNA728296	FJ626214.1	Iridian genomes
*Neoarius aff. graeffei *	SRR14629391	PRJNA708331	FJ013173.1	Iridian genomes
*Neoarius paucus*	SRR22495387	PRJNA837925	FJ626214.1	Iridian genomes
*Neoarius pectoralis*	SRR22495389	PRJNA837297	FJ013173.1	Iridian genomes

SRA, Sequence Read Archive.

## Supplementary Materials

Supplementary Material 1. The mitochondrial genome of the ten species used in phylogenetic reconstruction with GenBank accession number. The tree reconstruction included species belonging to the Ariidae family and three other more distantly related phylogenetic families.

Supplementary Material 2. All mitochondrial genomes of the eight species of Neoarius, along with their size and CG content.
