# Supplementary material for: Description of eight new mitochondrial genomes for the genus Neoarius and phylogenetic considerations for the family Ariidae (Siluriformes)
Source: Genomics Inform. 2023 Dec 29;21(4):e51. doi: 10.5808/gi.23059 (PMC10788360; doi:10.5808/gi.23059)
Supplement: Supplementary Material 1. — The mitochondrial genome of the ten species used in phylogenetic reconstruction with GenBank accession number. The tree reconstruction included species belonging to the Ariidae family and three other more distantly related phylogenetic families. [file gi-23059-Supplementary-Material-1.pdf]

Species	Genbank accession n°	Family
Arius arius	KX211965.1	Ariidae
Arius maculatus	MN604079.1	
Arius dispar	MH460877.1	
Occidentarius platypogon	KY930717.1	
Kryptoterus bicirrhis	NC_034999.1	Siluridae
Silurus asotus	MK895951.1	Siluridae
hemioliopterus	MF083116.1	Pimelodidae
melanodermatum	NC_065247.1	Pimelodidae
Hypostomus ancistroides	NC_052710.1	Loricariidae
Rineloricaria cf. lanceolata	KX087182.1	Loricariidae
