# Supplementary material for: Description of eight new mitochondrial genomes for the genus Neoarius and phylogenetic considerations for the family Ariidae (Siluriformes)
Source: Genomics Inform. 2023 Dec 29;21(4):e51. doi: 10.5808/gi.23059 (PMC10788360; doi:10.5808/gi.23059)
Supplement: Supplementary Material 2. — All mitochondrial genomes of the eight species of Neoarius, along with their size and CG content. [file gi-23059-Supplementary-Material-2.pdf]

Species	Size	CG Content
Neoarius utarus	16702bp	44%
Neoarius graeffei	16709bp	44%
Neoarius midgleyi	16709bp	45%
Neoarius leptaspis	16709bp	45%
Neoarius berneyi	16709bp	45%
Neoarius aff. graeffei	16710bp	45%
Neoarius paucus	16709bp	45%
Neoarius pectoralis	16711bp	44%
